# Antibiotic consumption and resistance of gram-negative pathogens (collateral damage)

**DOI:** 10.3205/id000040

**Published:** 2018-08-09

**Authors:** Milan Cižman, Tina Plankar Srovin

**Affiliations:** 1 University Medical Center, Department of Infectious Diseases, Ljubljana, Slovenia

## Abstract

Antibiotics are commonly prescribed in community and hospital care. Overuse and misuse favors emergence and spread of resistant bacteria. The ATC/DDD methodology is commonly used for presenting the drug utilization data. In primary care, the consumption is usually expressed in DDD per 1,000 inhabitants per day, in hospital, preferably in DDD per 100 bed days and DDD per 100 admissions. The alternative metric is days of therapy (DOT), which needs IT support. Antibiotics have ecological adverse effects at individual and population level. Antibiotics select resistant bacteria among pathogens and normal flora. Broad-spectrum antibiotics, low dosage and prolonged antibiotic therapy favor the development of resistance. Although total use of antibiotics in hospital is much less than in the community, the intensity of use magnified by cross infection ensures a multitude of resistant bacteria in today’s hospitals. Reversal of resistance is complex and might persist for many years despite the introduction of antimicrobial containment and stewardship programs.

## Summary of recommendations

1. Antimicrobial selective pressure allows microorganisms with intrinsic or newly acquired resistance to survive and proliferate. Use, misuse, or overuse of antimicrobial drugs are a major driving force towards antimicrobial resistance in the community and in hospitals. To minimize the selection pressure of antimicrobials it is fundamental to optimize antimicrobial use.
2. Fluoroquinolones, 3^rd^ generation cephalosporins, and carbapenems have greater impact on the emergence of resistance of Gram-negative bacteria compared to aminoglycoside and extended-spectrum penicillins with β-lactamase inhibitors. The urologic community can fight resistance development by ensuring the appropriate use of antibiotic prophylaxis and treatment.
3. Ciprofloxacin should not be used as the first line treatment for uncomplicated urinary tract infections due to a high risk of developing antibiotic resistance and collateral damage on gut microflora, which is the main reservoir of bacteria which causes UTI.
4. Fluoroquinolones generate resistance to the other antimicrobial drugs including carbapenems as well.
5. High-dose and shorter duration of antibiotic regimens may reduce the pressure on the emergence of antibiotic resistance in the community and in hospitals.

## 1 Introduction

Antimicrobial agents, predominantly antibiotics (antibacterials), are commonly prescribed in ambulatory and hospital care. The data from ECDC show that every day from 1 to over 3% of outpatients and 20 to 50% of inpatients are treated with antibiotics, with even higher percentage in intensive care units (ICUs) and hematology-oncology departments [1], [2], [3]. Antibiotics are also commonly (1–19%) prescribed in long-term facilities (nursing homes) [4]. Approximately 90% of all antibiotics in human medicine are used in outpatients and 85% of all hospital used antibiotics are prescribed at non-ICU wards [1], [5]. Unnecessary and inappropriate use of antibiotics favors emergence and spread of resistant bacteria. Up to half of all antibiotics use is thought to be inappropriate [6].

Antibiotic resistance is a consequence of antibiotic use. Bacteria adapt to the threat of antibiotics using mechanisms to overcome the drug. These resistant bacteria then survive. Bacteria evolve resistance to antibiotics by one of two routes; spontaneous mutations and horizontal gene transfer. In the presence of antibiotics, resistant mutants quickly outnumber sensitive bacteria and thus rapidly spread through a population eventually rendering the drug ineffective [7].

Antibiotics affect both the pathogens and a normal flora. Human gut and oral communities are recognized as reservoirs for the evolution and horizontal transfer of antibiotic resistance determinants including to pathogens. In theory, any antibiotic can select resistant strains as the local concentration of the drug exceeds the minimal inhibitory concentration (MIC) for the susceptible bacterial population but is below the MIC for the resistant clone. To what extend disturbances occur depends on the spectrum of the agent, dose, route of administration, pharmacokinetic, and pharmacodynamic properties, and in vivo inactivation of the agent. Not all antibiotics are likely to be equally selective for resistance [8]. Some antibiotics (e.g. fluoroquinolones) induce resistance to other antibiotic classes. Time to develop resistance is different as well. The selection of mutational resistance is often promoted by prolonged therapy, by infection sites where it is difficult to achieve high drug concentrations, and by under-dosage [8]. There is a perception that antimicrobial-resistant microbes might be less fit (i.e. less able to grow or cause an infection) than their antimicrobial susceptible counterparts [9]. Unfortunately, this is frequently not the case. Worldwide clonal spread and long-term persistence of resistant bacteria are also seen in the absence of direct antibiotic selection pressure [9]. It is particularly worrisome that the resistance is easy to acquire but once it develops, antimicrobial resistance is either irreversible or very slow to reverse, despite the introduction of antimicrobial resistance containment and stewardship programs.

Collateral damage is a term to refer to ecological adverse effects of antibiotics therapy at individual and population level; that is the selection of antibiotic-resistant organisms and *Clostridium difficile* [10].

Among Gram-negative bacteria which most often cause urinary tract infections (UTI) and represent increasing resistance problem are Enterobacteriaceae and the non-fermenters *P. aeruginosa *and *Acinetobacter baumannii*. Distribution of resistance types in hospitals, long-term facilities, and the community is increasingly blurred with many elderly patients moving back and forth between hospital and care home.

The purpose of the review is to show the correlation between antibiotic usage and resistance of Gram-negative bacteria.

## 2 Methods

**Figure 1:**
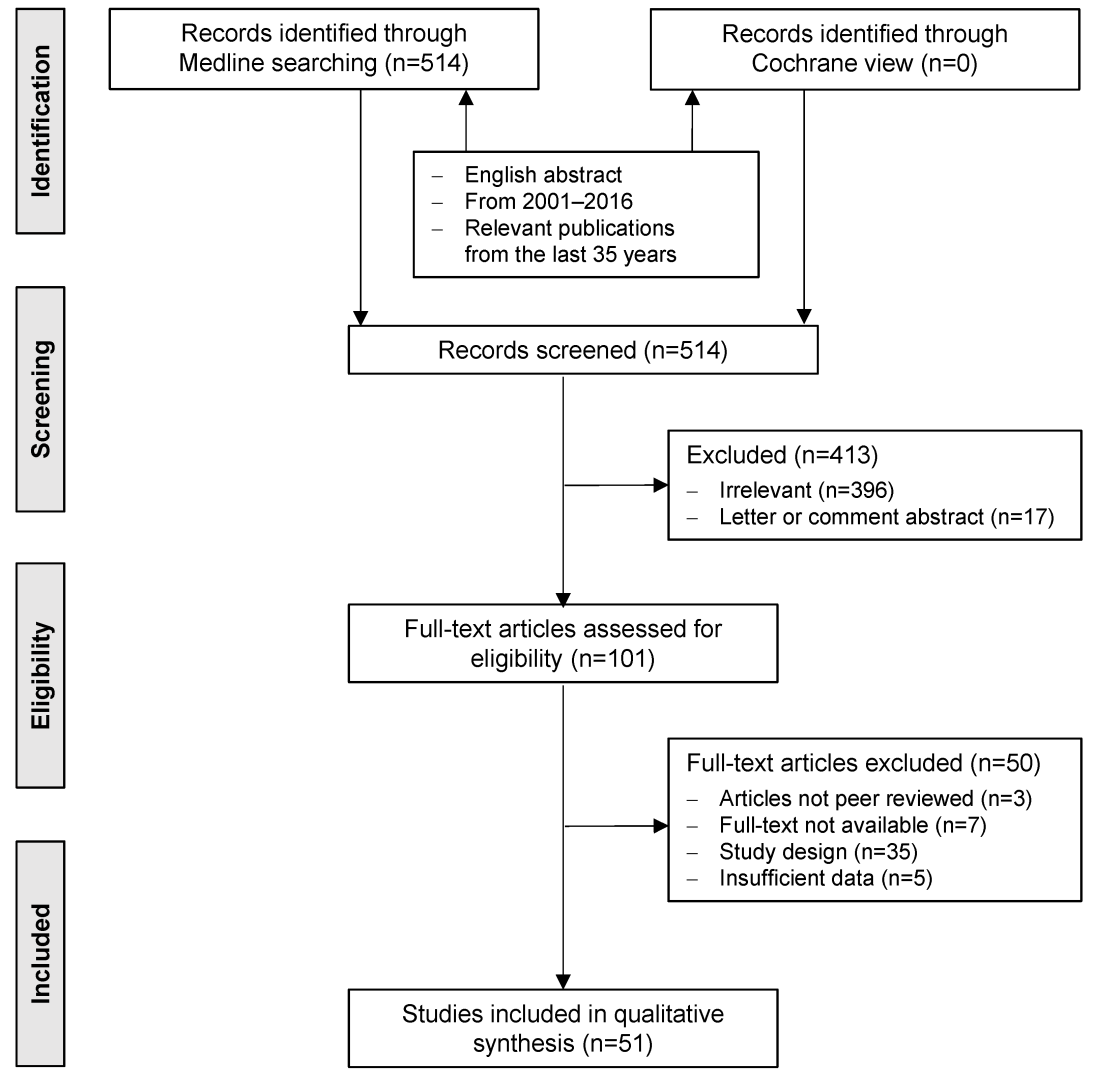
PRISMA flow diagram of the systematic review [63]

A systematic literature search was performed for the last fifteen years, from 2001 to 2016, in Medline and Cochrane library with the following key words: measurement of antibiotic consumption; antibiotic consumption and antibiotic resistance and (Gram-negative or *E. coli* or *Klebsiella *spp. or *Proteus *spp. or *Pseudomonas *spp. or *Serratia* spp. or *Acinetobacter* spp.); reversal of antibiotic resistance. We also considered other relevant publications from the last 35 years. An English abstract had to be available. A total of more than 500 publications were identified: 87 for the measurement of antibiotic consumption, 324 for the antibiotic consumption and antibiotic resistance in different Gram-negative bacteria, and 103 for the reversal of antibiotic resistance. No Cochrane review was published. The publications were screened by title and abstract. Finally, 51 publications were included into this review (Figure 1 (Fig. 1)).

The studies were rated according to the level of evidence (LoE) and the strength of recommendations (GoR) graded according to a system used in the EAU guidelines (2015) modified from the Oxford Centre for Evidence-based Medicine ([11] p. 3 Methodology section).

## 3 Results

### 3.1 Measurement of antibiotic consumption

The anatomical therapeutic chemical (ATC) defined daily doses (DDD) methodology is commonly used as the method for presenting the drug utilization data [12]. The DDD is defined as the assumed average maintenance dose per day for a drug used for its main indication in adults. As the dosages used may change over time, the DDD should be periodically updated. The DDD is an international unit that can be used for comparisons of antibiotic use. In primary care, the consumption is usually expressed in number of DDDs per 1,000 inhabitants per day (DID) [12]. Without changes of DDDs per package (PID) over time, we found a good correlation between DID, PID and number of prescriptions per 1,000 inhabitants per year in contrast to Belgium where changes of packages were seen over time (LoE 2a) [13], [14].

Hospital drug use should preferably be presented as DDDs per 100 bed days/occupied bed days and DDDs per 100 admissions [12], [15], [16]. The WHO-defined DDD has also been used to demonstrate a quantitative, ecological relationship between antimicrobial use and resistance in hospitals [17]. The disadvantages of DDD are that it may not reflect the prescribed daily dose (PDD) for individual patients, it cannot be used to measure consumption in pediatric wards since the measure is based on adult dosing, and it does not accurately measure antibacterial consumption in cases of renal or hepatic dysfunction often underestimating actual antibacterial usage [18]. The alternative metrics to measure antibiotics use in hospitals include the recommended daily dose (RDD), PDD, days of therapy (DOT), length of therapy (LOT) per admissions and per patients, patients exposed to antibiotics per all patients and per admissions and DDD per 100 bed days per case mix index [19], [20] (LoE 2a; GoR B).

In summary, the “best” measurement of antibiotic use is still under investigation. Furthermore, antibiotic use, as discussed about, is not necessarily the same as antibiotic exposure [19]. The exposure of antibiotics at the individual level (dosing regimen, duration of therapy) is an important factor in the selection of antibiotic-resistant mutants.

### 3.2 Development of resistance – patient level

Patients receiving antibiotic therapy often experience the increase of resistant bacteria as is well documented by a number of prospective studies (LoE 1a) [21]. Careful analysis of prospective studies that included large number of patients showed that the mean resistance rates during monotherapy ranged from 4.7% to 13.4% [22]. In accordance with animal model, *Pseudomonas aeruginosa* appeared particularly to be able to produce resistance during antibiotic therapy [22].

Emergence of resistance during therapy also depends on the drug administered to the patient. For example, streptomycin has only a single ribosomal binding site and is compromised by high frequency mutations that alter this site; other aminoglycosides have multiple binding sites and are not compromised so easily [8]. The study from Gerding et al. indicated little impact of the aminoglycosides on the susceptibility of bacteria to other agents but a clear effect of drug consumption in resistance within their own class of antibiotics (LoE 2a) [23]. Quinolone resistance is mostly caused by chromosome mutations [8]. Fluoroquinolones can also select for mutants of Gram-negative bacteria that overproduce a wide variety of efflux pumps and in a single step cause resistance to practically all classes of antimicrobial agents including carbapenems in *P. aeruginosa* (LoE 1a) [24]. Three-days course of ciprofloxacin for treatment of uncomplicated cystitis was followed by isolation of fluoroquinolone-resistant *E. coli* in one out of 25 treated patients. No emergence of resistance was observed in the 7 days of nitrofurantoin therapy or a single dose of fosfomycin [25]. Even a low rate of selection pressure could translate high usage of fluoroquinolones into substantial population-based rates of fluoroquinolones resistance.

Second- and third-generation cephalosporins are associated with the selection of resistant Enterobacteriaceae via acquisition of extended-spectrum β-lactamases and in the case of *Enterobacter* spp., *Citrobacter* spp., *Morganella* spp., and *Serratia* spp. with selection of mutants that hyperproduce chromosomal AmpC β-lactamases. These mutants are frequently selected during therapy causing clinical failure in individual patients [8]. Fourth-generation cephalosporins (cefepime and cefpirome) are more stable to AmpC β-lactamases than third-generation analogues and therefore less selective for AmpC derepressed mutants (LoE 1a) [8].

Imipenem selected resistance in about 5% of patients almost solely in *P. aeruginosa* infections [21]. Hong et al. documented the first case of carbapenem-resistant *E. coli* emergency during therapy with imipenem and meropenem and first identified carbapenem-hydrolyzing enzyme in *E. coli* isolates [26]. The systematic review and meta-analysis showed that carbapenem use was the leading risk factor, odds ratio (OR) 7.09; 95% confidence interval (CI) 5.43–9.25 for carbapenem-resistant *Pseudomonas aeruginosa* (LoE 1a) [27].

Recent systematic review and meta-analysis showed that the exposure in primary care was consistently associated with a subsequent twofold risk of antibiotic resistance in respiratory and urinary bacteria for up to 12 months after the treatment. Longer duration and multiple courses were associated with higher rates of resistance (LoE 1a) [28].

### 3.3 Previous antibiotic therapy

Previous antibiotic therapy is well known risk factor for colonization and infection with resistant bacteria [9]. Ortega et al. found out that previous beta-lactam antibiotic therapy doubled the risk (OR 1.68) for acquiring ESBL-producing *E. coli* bloodstream infection, whereas previous quinolone therapy was associated with even higher risk of fluoroquinolone-resistant *E. coli* (OR 7.41) [29]. Independent risk factors for fluoroquinolone resistance in a nested case-control study in adults with community-onset urinary tract infections were urinary catheter (OR 3.1), recent hospitalization (OR 2.0), and fluoroquinolone use in the past 6 months (OR 17.5; 95% CI 6.0–50.7) [30]. Dan et al. reported that prior use of fluoroquinolones within 90 days (adjusted odds ratio (aOR) 7.87; 95% CI 4.53–13.79) or within 91–180 days (aOR 2.77; 95% CI 1.17–6.16) was an independent risk factor for bloodstream infections with fluoroquinolones-resistant Gram-negative bacteria. The predicted probability of fluoroquinolone resistance was 39% and 22%, respectively [31].

In a prospective cohort study conducted in ambulatory care, Stewardson et al. found that ciprofloxacin had a significant global impact on the gut microbiota whereas nitrofurantoin did not. This study supports the use of nitrofurantoin over fluoroquinolones for treatment of uncomplicated urinary tract infections to minimize perturbation of intestinal microbiota. Substantial recovery had occurred 4 weeks later [32].

A recent case-control study showed that the risk for ampicillin resistant *E. coli* infection in 903 patients was associated with amoxicillin prescription ≥7 days duration in the previous month (OR 3.91; 95 % CI 1.64–9.34) and previous 2–3 months (OR 2.29; 95% CI 1.12–4.70) before illness onset [33]. For treatment <7 days, there was no statistically significant association. Higher doses of amoxicillin were associated with a lower risk of ampicillin resistance. For trimethoprim-resistant *E. coli* infections, the OR was 8.44 (95% CI 3.12–22.86) for duration of trimethoprim treatment ≥7 days in the previous month and 13.91 (95% CI 3.32–58.31) for the previous 2–3 months. For trimethoprim prescriptions of <7 days, the OR was 4.03 (95% CI 1.69–9.59) for the previous month, but treatment in earlier periods was not significantly associated with resistance. The study showed that high-dose, shorter-duration antibiotic regimens may reduce the pressure on the emergence of antibiotic resistance (LoE 2a) [33].

### 3.4 Development of resistance – community level

The relationship between antibiotic prescribing in the community and resistance is well characterized (LoE 2a) [34]. The causal relationship between antibiotic use and resistance defining and quantifying this for a given antibiotic and given resistance are extremely difficult. In particular, it is difficult to control for all the confounding factors that play a role in the development and spread of resistance [9], [35].

Confounding factors for development of resistance [9]:

- Pathogen-drug interaction
- Pathogen-host interaction
- Mutation rates of the pathogen
- Emergence of successful antimicrobial resistant clones
- Transmission rates of pathogens (between human beings, animals, environment)
- Cross-resistance
- Co-resistance
- Public health factors (vaccine uptake, health care system)
- Migration, tourism
- Sanitation
- Population densities

There are some countries with a relatively high percentage of resistance despite lower consumption, which suggests that factors other than the national antimicrobial consumption in humans may contribute (LoE 1a) [24], [36].

In a systematic review and meta-analysis of 243 studies, Bell et al. reported an association between antibiotic consumption and the subsequent development of bacterial resistance at both the individual and community level (LoE 1a) [37]. Increased consumption of antibiotics may not only produce greater resistance at the individual patient level but may also produce greater resistance at the community, country, and regional level which can harm individual patients.

According to the ESAC-Net 2012, the total (hospital and community) consumption of fluoroquinolones between 0.5–1.2 DID and community consumption between 0.4–0.9 DID was associated with a low prevalence (11–15%) of fluoroquinolone resistant invasive *E. coli* in 2013. In countries with a high prevalence of fluoroquinolones resistant *E. coli* (40–52%), the total and community fluoroquinolone consumption was between 2.3–3.84 DID and 2.0–3.4 DID, respectively [1], [38]. Van de Sande-Bruinsma et al. found a reasonable correlation (correlation coefficient R 0.60 (0.44–0.70) between use of fluoroquinolones in European countries in the period 2002–2004 and resistance of invasive strains of *E. coli* in the period 2002–2005 (LoE 2a) [39].

In joint ECDC/EFSA/EMA report, the authors found a significant association between resistance of *E. coli* in humans to 3^rd^ generation cephalosporins and consumptions of 3^rd^ and 4^th^ generation cephalosporins in the community, in hospitals, and in total (LoE 2a) [40].

As Bergman et al. noted, population-level antibiotic pressure may have more effect on an individual risk for resistant organisms than individual antimicrobial usage [41]. Therefore, unnecessary use of antibiotics, particularly broad-spectrum, should be avoided (LoE 2a; GoR B).

### 3.5 Development of resistance – hospital level

Although the total use of antibiotics in hospitals is much less than in the community, the intensity of use, magnified by cross infection, ensures a multitude of resistant bacteria in today’s hospitals. The degree of antimicrobial resistance in these settings depends on antimicrobial consumption, intrinsic factors including infection control, and on external factors such as influx of resistant pathogens [42]. Antimicrobial consumption also shows large inter-hospital differences that might be explained by case-mix, antibiotic stewardship, infection control, and other factors. There are numerous examples to illustrate the relation between antimicrobial use and antimicrobial resistance. A statistically significant correlation was identified between the rates of Enterobacteriaceae resistant to 3^rd^ generation cephalosporins, particularly those secreting β-lactamases with extended-spectrum and consumption of 3^rd^ generation cephalosporins (LoE 2a) [40], [43]. Increased use of fluoroquinolones was correlated with a higher incidence of ciprofloxacin resistance in Gram-negative bacilli (LoE 2a) [40], [44]. A strong association between carbapenem consumption and carbapenem resistance in Gram-negative bacteria was also reported (LoE 1a) [27], [45]. However, the relation between consumption and resistance is complex and different results have been reported as well (LoE 2a) [46], [47].

Ascioglu et al. simultaneously analysed individual- and population-level determinants of bacterial resistance in the hospital [49]. For Gram-negative bacteria, the use of the major selecting antibiotic by an individual was the main risk factor for acquiring resistant species. Hazard ratios (HRs) were strikingly high for ceftazidime-resistant *Enterobacter* spp. (HR 11.17; 95% CI 5.67–22.02), ciprofloxacin-resistant *P. aeruginosa* (HR 4; 95% CI 2.14–9.08) and imipenem-resistant *P. aeruginosa* (HR 7.92; 95% CI 4.35–14.43). Ward level use was significant for imipenem-resistant *P. aeruginosa* (HR 1.22; 95% CI 1.15–1.30). Their results showed that combining population- and individual-level data is crucial for the exploration of antimicrobial resistance development (LoE 1b) [48], [49].

### 3.6 Reversal of resistance

If the usage of antimicrobials is the main driver of resistance, it seems logical to reduce resistance by reducing usage of them. Evidence of positive correlations are mixed (LoE 1a) [8], [50], [51], [52], [53], [54]. In the UK the, consumption of sulfonamides decreased by 97% from 1991 to 1999. In the same period, the prevalence of sulfonamide resistance in urinary *E. coli* in London rose from 39.2% to 45.8% [53]. In Kronoberg County, Sweden, the use of trimethoprim containing drugs decreased by 85% over 24 months. Segmented regression analysis could not detect any significant trend-break in the trimethoprim resistance rate in *E. coli* in relation to the intervention (LoE 2a) [54]. Austin et al. proposed the threshold effect in the late 1990s, which suggests that resistance will occur at some time-point, once the corresponding antibiotic or antibiotic class consumption exceeds a specific threshold [55]. In general, based on observations in Finland and Iceland, it appeared that once use exceeds 15–25 DID in a population or 15–25 DDD/1,000 bed days in hospital settings, the resistance will occur (LoE 2a) [55]. To reverse the resistance, a reduction of prescribing by at least 50% would be needed to achieve levels below the antimicrobials agent’s resistance threshold (Table 1 (Tab. 1)). Recently, Sarma et al. published that a significant decrease in ciprofloxacin resistance of *E. coli* ESBL-producing urinary isolates in hospitals and in the community was observed, within four months after the hospital reduction of fluoroquinolone use ≤2 DDD/100 occupied bed days and after a 46% decline of monthly number of fluoroquinolone prescriptions in the community. The decline was also marked in all urinary isolates of Enterobacteriaceae and *E. coli* isolates from blood cultures (LoE 2a) [56]. The adherence to European Association of Urology guidelines on antibiotic prophylaxis for surgical urologic procedures reduced overall antibiotic consumption and the risk of resistance development among uropathogens without increasing the risk of postoperative infection and with reduced health care costs (LoE 2a) [57]. During the study period, the consumption of ciprofloxacin decreased significantly (p<0.001) from 4.2 to 0.2 DDD/100 patient days and the use of aminoglycosides from 1.5 to 0.4 DDD/100 bed days (p<0.001). The rate of resistance of *Escherichia coli* to gentamicin (18.3% vs. 11.2%; p=0.02) and ciprofloxacin (32.3% vs. 19.1%; p=0.003) decreased significantly after protocol introduction (LoE 2a) [57].

In recent years, many hospitals introduced an antibacterial stewardship program, which was followed by a decreased use of antimicrobials and different effects on the reversal of Gram-negative bacteria resistance in health care settings (LoE 2a) [58], [59], [60]. The urologic community can fight resistance development by ensuring the appropriate use of antibiotic prophylaxis and treatment (GoR B) [61].

## 4 Further research

The best measurement unit for measuring antimicrobial consumption is under investigation. The optimal total and pattern of antimicrobial use in the community and hospital care should be determined. The specific threshold of antimicrobial consumption for development of resistance should be determined as well. The correlation between antibiotic use and antibiotic resistance is complex and further studies are needed.

## 5 Conclusions

The appropriate and particularly inappropriate use of antimicrobials is considered to be a major driving force towards antimicrobial resistance. Antimicrobial use exerts selective pressure on commensal human microflora and pathogens, increasing the risk of recovery of resistant organisms from patients. Patients receiving and/or having received antibiotics in recent past often experience the risk of acquiring resistant bacteria. The role of antimicrobial use in driving the emergence of resistance is likely to be specific to each drug and to each microorganism, as is the effect of the increase or reduction in this use. To reduce antimicrobial resistance the combining population- and individual-level consumption should be optimized.

## Note

This article is also to be published as a chapter of the Living Handbook “Urogenital Infections and Inflammations“ [62].

## Competing interests

The authors declare that they have no competing interests.

## Figures and Tables

**Table 1:**
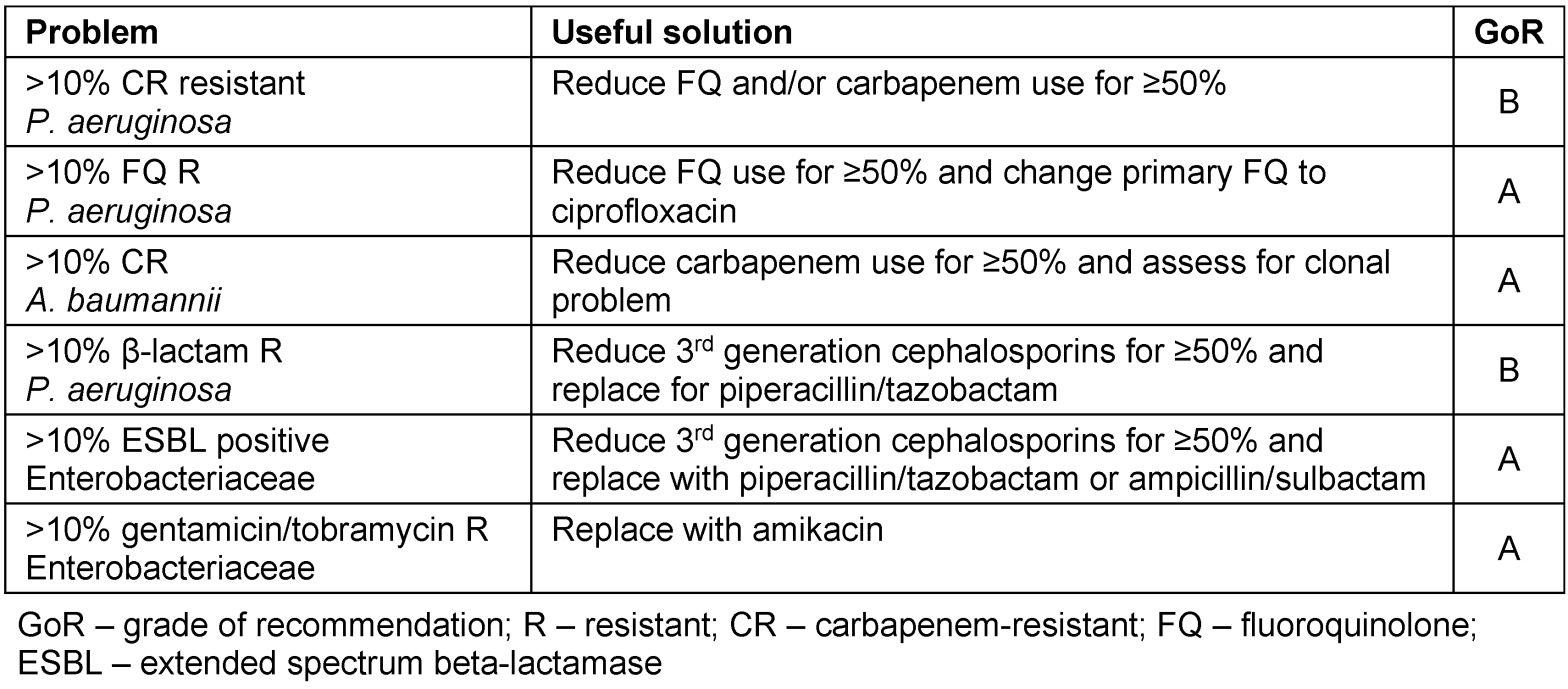
Recommendations for management of antimicrobial agent resistance – the goal of using less than 1.5 DDD/100 patient days [24]
